# Hydrogen Peroxide-Mediated Inhibition of Membrane Resealing Drives Synergistic Cytotoxicity of Combined Cold Atmospheric Plasma and Pulsed Electric Field Treatment

**DOI:** 10.3390/ijms27062700

**Published:** 2026-03-16

**Authors:** Seiji Kushibiki, Hirofumi Kurita

**Affiliations:** Department of Applied Chemistry and Life Science, Toyohashi University of Technology, Toyohashi 441-8580, Aichi, Japan

**Keywords:** cold atmospheric plasma, plasma medicine, oxidative stress, hydrogen peroxide, electroporation, pulsed electric field, membrane permeabilization

## Abstract

Cold atmospheric plasma (CAP) combined with pulsed electric fields (PEF) demonstrates synergistic cytotoxicity against HeLa cells; however, the differential contributions of short-lived versus long-lived reactive oxygen and nitrogen species (RONS) remain unclear. This study compared direct CAP treatment with indirect CAP-treated liquid treatment, both followed by PEF, to elucidate underlying mechanisms. Direct CAP + PEF treatment resulted in significantly greater cell death than indirect CAP + PEF, with both showing synergistic effects relative to single treatments. Analysis of intracellular RONS and membrane integrity revealed that direct CAP treatment enhanced intracellular RONS levels and PEF-induced membrane permeabilization immediately after treatment. Time-course analysis demonstrated that hydrogen peroxide specifically inhibits membrane resealing following PEF-induced electroporation, as evidenced by progressive calcein leakage over 20 min, while immediate pore formation remained unaffected. Catalase rescue experiments confirmed that hydrogen peroxide removal prevented progressive membrane damage without affecting immediate pore formation, thereby restoring cell viability. These findings identify hydrogen peroxide-mediated inhibition of membrane resealing as a novel mechanism underlying synergistic cytotoxicity, distinct from immediate membrane damage. This two-phase mechanism provides new insights for optimizing plasma-based cancer therapies.

## 1. Introduction

Interest in cold atmospheric plasma (CAP) applications in life sciences, medicine, and agriculture has increased significantly in recent decades. CAP treatment has been extensively studied for diverse therapeutic applications, including cancer therapy, wound healing, blood coagulation, dentistry, and the prevention of infectious diseases [1,2,3,4,5]. Additionally, CAP has emerged as a promising approach for delivering cell-impermeable molecules into living cells [6,7,8,9], demonstrating its versatility as a technique for applications such as gene transfection in life sciences and medical research. The biological effects of CAP are primarily mediated by reactive oxygen and nitrogen species (RONS), whose spatiotemporal distribution is a critical determinant of biological dose [10,11,12]. Short-lived RONS such as hydroxyl (OH) radicals and superoxide are highly reactive and decay within microseconds or less, confining their activity to the immediate vicinity of CAP. Long-lived RONS, including hydrogen peroxide (H_2_O_2_), nitrite (NO2^−^), and nitrate (NO3^−^), remain stable in aqueous solutions for extended periods, enabling spatial diffusion and temporal persistence. Consequently, the composition of RONS delivered to cells differs fundamentally between direct CAP treatment, which delivers both short-lived and long-lived species, and indirect CAP treatment using plasma-treated liquids, which primarily delivers long-lived species. While the mechanisms underlying CAP effects are being progressively elucidated, a more comprehensive understanding is required to establish CAP treatment as a safe and reliable therapeutic tool.

To enhance efficacy and selectivity, combination approaches integrating CAP with other modalities have attracted considerable attention. These strategies are particularly promising because CAP induces sublethal cellular alterations—including DNA damage, protein oxidation, and cellular senescence—even at low doses that maintain cell viability [13,14,15,16,17]. While such low-dose CAP treatments alone exhibit limited efficacy, they create exploitable cellular vulnerabilities that can be leveraged through combination with complementary treatments to achieve enhanced effects while minimizing direct cytotoxic damage.

Various combination modalities have demonstrated synergistic effects [18,19]. Chemical approaches using nanoparticles show promise for tumor-selective delivery of therapeutic agents [20], while chemotherapeutic drugs such as doxorubicin and cisplatin demonstrate enhanced cytotoxicity when combined with CAP [21,22,23,24,25]. Physical modalities offer complementary mechanisms: hyperthermia [26,27,28] and ultrasound [29,30] enhance membrane permeability through thermal or mechanical effects, whereas pulsed electric fields (PEF) provide precise temporal control of membrane permeabilization.

PEF application creates transient pores that increase membrane permeability. This phenomenon, known as electroporation or electropermeabilization, enables delivery of cell-impermeable molecules, such as DNA, RNA, proteins, and small molecules, into cells; therefore, PEF is widely used for gene electrotransfer in vitro and in vivo [31,32,33]. They have also been applied in electrochemotherapy for delivering chemotherapeutic agents into cells [34,35]. Recent studies have explored the potential synergistic effects of combining CAP treatment with PEF application, with several research groups reporting promising results. Jiang et al. demonstrated synergistic effects of nanosecond pulsed plasma jets and PEF on both cancer cell death in vitro and plasmid DNA delivery in vivo [36,37]. Similarly, Wolff et al. reported enhanced intracellular RONS accumulation and immunogenic cell death in leukemia cells following combined treatment [38]. Chung et al. investigated the combined effects of indirect CAP treatment using CAP-treated phosphate-buffered saline with microsecond PEF [39].

Our previous investigation demonstrated enhanced cytotoxicity in HeLa cells following combined CAP and PEF treatment [40]. We showed that CAP pretreatment sensitized cells to subsequent PEF-induced electroporation, facilitating intracellular uptake of CAP-generated long-lived RONS. Furthermore, we found that CAP pretreatment enhanced membrane permeabilization induced by PEF. To further elucidate the enhanced cytotoxicity, this study compared the cytotoxic effects of direct CAP irradiation with indirect treatment with CAP-treated liquid, both followed by PEF application. This comparison aims to elucidate the distinct mechanisms underlying each treatment approach and identify the key factors contributing to the synergistic effects. Since direct treatment involves both short-lived and long-lived RONS, while indirect treatment primarily involves long-lived species, this comparison will help clarify the role of different reactive species in the synergistic effects.

## 2. Results

**Figure 1:**
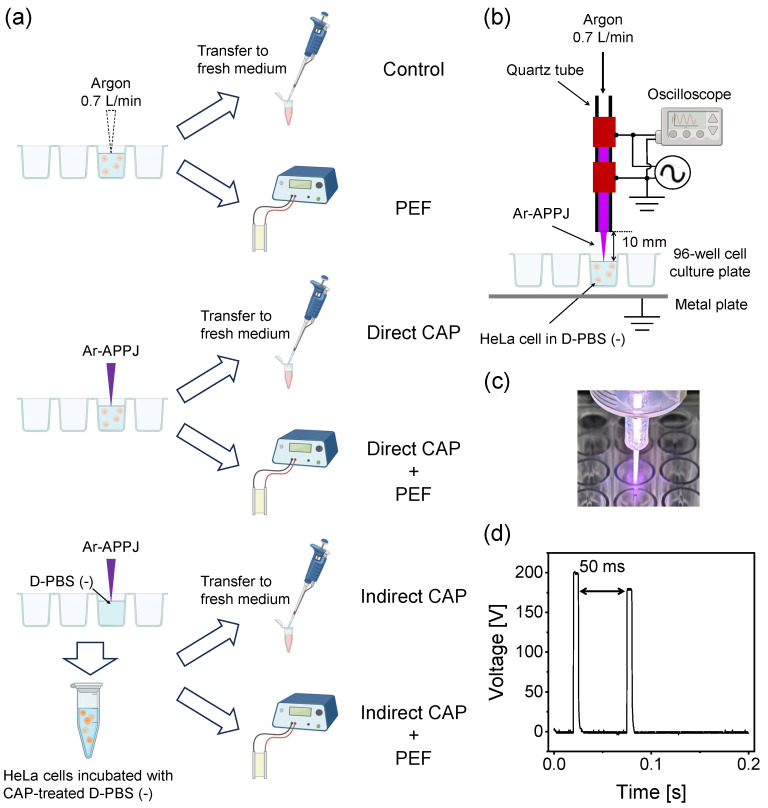
Experimental setup and treatment procedure. (**a**) Treatment protocol flowchart for all experimental groups tested in this study. Six experimental groups were compared: Control (Ar gas flow only), PEF alone, Direct CAP (Ar-APPJ irradiation directly onto cell suspensions), Direct CAP + PEF, Indirect CAP (cells incubated in CAP-treated D-PBS), and Indirect CAP + PEF. Direct treatment delivers both short-lived and long-lived RONS, while indirect treatment primarily delivers long-lived RONS. (**b**) Schematic illustration of the Ar-APPJ irradiation setup showing the plasma jet generator positioned 10 mm above the cell suspension or cell-free D-PBS (−) in a 96-well plate. The generator consists of a quartz glass tube with copper tape electrodes. (**c**) A photograph of the Ar-APPJ (reproduced from [40] under CC BY 4.0). (**d**) Pulse waveform for PEF application. Two consecutive pulses (5 ms duration, 50 ms interval, 1.0 kV/cm maximum field strength, 10% decay ratio) were applied using a 2 mm-gap electroporation cuvette. Graphics were created with BioRender.com.

This study investigated the synergistic cytotoxicity of combined CAP and PEF treatment by comparing two distinct CAP treatment modalities. Figure 1 shows the experimental setup and treatment procedure. As illustrated in Figure 1a, direct CAP treatment involved argon atmospheric pressure plasma jet (Ar-APPJ) irradiation (Figure 1b,c) directly onto HeLa cell suspensions, delivering both short-lived RONS and long-lived RONS. In contrast, indirect CAP treatment involved Ar-APPJ irradiation of cell-free D-PBS (−), followed by cell incubation in the CAP-treated D-PBS (−), primarily delivering long-lived RONS. Both treatment modalities were followed by PEF application (Figure 1d) with a 1-min interval between CAP and PEF treatments.

### 2.1. Cell Viability

**Figure 2:**
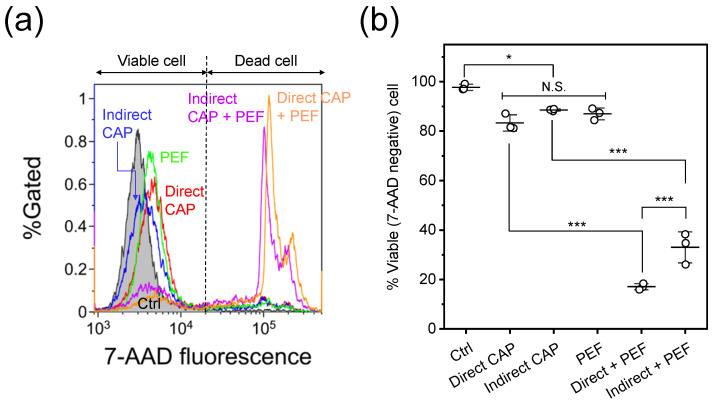
Flow cytometric analysis of cell viability 24 h after treatment. (**a**) Representative flow cytometry histograms showing 7-AAD fluorescence intensity. HeLa cells were assayed based on uptake of 7-AAD, a cell-impermeable dye that stains dead cells. The dashed line represents the threshold for distinguishing viable (7-AAD-negative) and dead (7-AAD-positive) cells. Control (Ctrl) indicates cells exposed to Ar gas flow for 3 min without plasma discharge. (**b**) Quantification of cell viability, defined as the percentage of 7-AAD-negative cells. Data are expressed as mean ± standard deviation (SD) (n=3). Statistical significance was determined using one-way ANOVA followed by Tukey’s multiple comparison test. * p<0.05 and *** p<0.001. N.S., not significant.

Figure 2 shows cell viability 24 h after treatment. Treated HeLa cells were stained with 7-AAD and analyzed by flow cytometry. Figure 2a shows representative flow cytometry histograms, with 7-AAD-negative cells defined as viable. Significant increases in 7-AAD-positive cell populations were observed in both combined treatments. Figure 2b shows quantitative cell viability obtained from the histograms. Each single treatment (direct CAP, indirect CAP, or PEF) significantly decreased viable cell populations compared to control (p<0.05); however, viability remained above 80% for all single treatments, with no significant differences among them (N.S.). Both combination treatments showed statistically significant decreases in viability compared to their respective single treatments (p<0.001). Direct CAP + PEF treatment showed significantly greater cytotoxicity (approximately 17% viability) than indirect CAP + PEF (approximately 33% viability; p<0.0001), demonstrating synergistic cytotoxic effects under these experimental conditions.

### 2.2. Intracellular RONS Measurement

**Figure 3:**
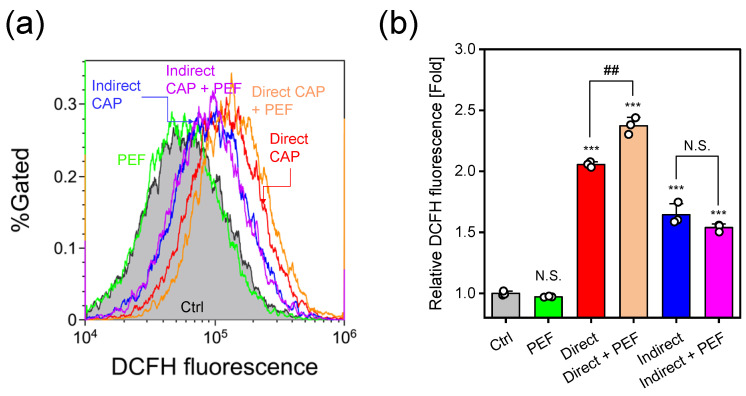
Intracellular RONS levels measured using photo-oxidation resistant DCFH-DA fluorescence. (**a**) Representative flow cytometry histograms of DCFH fluorescence intensity. (**b**) Quantitative analysis of relative DCFH fluorescence (normalized to control). Data are mean ± SD (n=3). Statistical significance was determined using one-way ANOVA followed by Tukey’s multiple comparison test for comparisons among different treatment groups, or paired *t*-test for comparisons between CAP alone and CAP + PEF from the same initial CAP-treated sample (direct CAP vs direct CAP + PEF; indirect CAP vs. indirect CAP + PEF). *** p<0.001 vs. control; ^##^ p<0.01 (paired *t*-test); N.S., not significant.

Figure 3 shows intracellular RONS levels assessed using photo-oxidation resistant DCFH-DA fluorescence probe and flow cytometry. Representative flow cytometry histograms are shown in Figure 3a. PEF treatment alone did not alter DCFH fluorescence intensity compared to control. Both direct and indirect CAP treatments significantly increased DCFH fluorescence, with direct CAP showing a greater increase than indirect CAP. Combined direct CAP + PEF treatment enhanced the fluorescence increase, whereas indirect CAP + PEF did not. Figure 3b shows quantitative comparison of relative median fluorescence intensity (MFI) normalized to control values. PEF treatment alone showed no significant change compared to control. Direct CAP treatment increased relative MFI approximately 2.06-fold (p<0.001), while indirect CAP showed a more modest increase to 1.64-fold (p<0.001). Notably, combined direct CAP + PEF treatment further enhanced MFI to 2.38-fold, significantly higher than direct CAP alone (p<0.01). In contrast, indirect CAP + PEF (1.54-fold) showed no significant enhancement compared to indirect CAP alone, resulting in lower fluorescence levels than direct CAP + PEF.

### 2.3. Lipid Peroxidation

**Figure 4:**
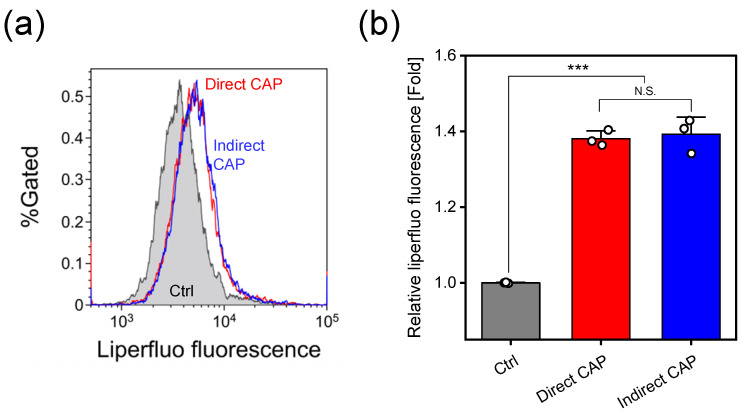
Lipid peroxidation in HeLa cells assessed using Liperfluo fluorescence. (**a**) Representative flow cytometry histograms of Liperfluo fluorescence intensity. (**b**) Relative median fluorescence intensity (MFI) normalized to control values. Data are mean ± SD (n=3). Statistical significance was determined using one-way ANOVA followed by Tukey’s multiple comparison test. Control (Ctrl): cells exposed to Ar gas flow for 3 min without plasma discharge. *** p<0.001; N.S., not significant.

Figure 4 shows lipid peroxidation assessed using Liperfluo fluorescence probe immediately after CAP treatment. Representative flow cytometry histograms (Figure 4a) demonstrate that both direct and indirect CAP treatments increased Liperfluo fluorescence intensity compared to control. Figure 4b shows quantitative analysis of relative median fluorescence intensity normalized to control values. Both direct and indirect CAP treatments significantly increased lipid peroxidation levels compared to control (direct CAP: approximately 1.38-fold; indirect CAP: approximately 1.39-fold; both p<0.0001). No significant difference was observed between direct and indirect CAP treatments (N.S.).

### 2.4. Calcein Leakage

**Figure 5:**
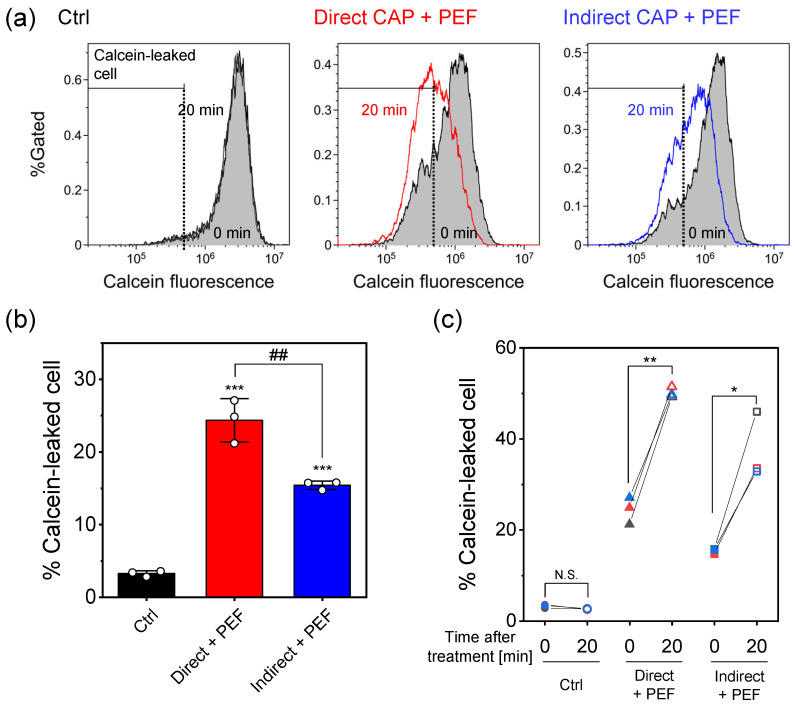
Measurement of calcein leakage assessed by flow cytometry. (**a**) Representative flow cytometry histograms of calcein fluorescence intensity. Control (Ctrl) represents cells exposed to Ar gas flow for 3 min without plasma discharge. The black dashed line indicates the threshold of the calcein-leaked cells. The flow cytometry histogram immediately after treatment is shown in gray. Colored histograms are 20 min after the first flow cytometry measurement. (**b**) Quantitative analysis of the percentage of calcein-leaked cells immediately after treatment. Data are mean ± SD (n=3). Statistical significance was determined using one-way ANOVA followed by Tukey’s multiple comparison test. *** p<0.001 vs. control; ^##^ p<0.01 (direct CAP + PEF vs. indirect CAP + PEF). (**c**) Time-course analysis comparing calcein leakage at 0 and 20 min. Circles, Ctrl; triangles, Direct + PEF; squares, Indirect + PEF. Each color represents an individual replicate from three independent experiments. Statistical significance was determined using paired *t*-test. * p<0.05; ** p<0.01; N.S., not significant.

Figure 5 shows calcein leakage assessed by flow cytometry. Figure 5a shows representative flow cytometry histograms, with overlays comparing measurements obtained immediately after treatment (filled gray) and 20 min later. The threshold for calcein-leaked cells was determined using the control experiment. Our previous report demonstrated that PEF alone induced a slight increase in the population of calcein-leaked cells, whereas direct CAP alone showed no significant change in the population of calcein-leaked cells. Based on these observations, indirect CAP alone was expected to show minimal calcein leakage; therefore, we focused on both direct CAP + PEF and indirect CAP + PEF treatments. Both combination treatments showed a significant increase in the population of calcein-leaked cells. In addition, both combination treatments dramatically increased the proportion of calcein-leaked cells 20 min after the initial flow cytometry measurement.

Figure 5b shows quantitative analysis of the percentage of calcein-leaked cells immediately after treatment. Both combination treatments significantly increased the percentage of calcein-leaked cells compared to control (p<0.001). Direct CAP + PEF showed significantly greater immediate membrane damage (approximately 24% calcein-leaked cells) than indirect CAP + PEF (approximately 15%; ** p<0.01).

Time-course analysis (Figure 5c) demonstrated progressive membrane damage in both combination treatments. The percentage of calcein-leaked cells increased significantly from 0 to 20 min in both direct CAP + PEF (from ∼24% to ∼50%; ** p<0.01, paired *t*-test) and indirect CAP + PEF (from ∼15% to ∼37%; * p<0.05, paired *t*-test), indicating sustained membrane disruption following the initial PEF-induced damage. Control cells showed no significant change over the same period (N.S.).

### 2.5. H_2_O_2_ Concentration and Catalase Rescue Experiments

**Figure 6:**
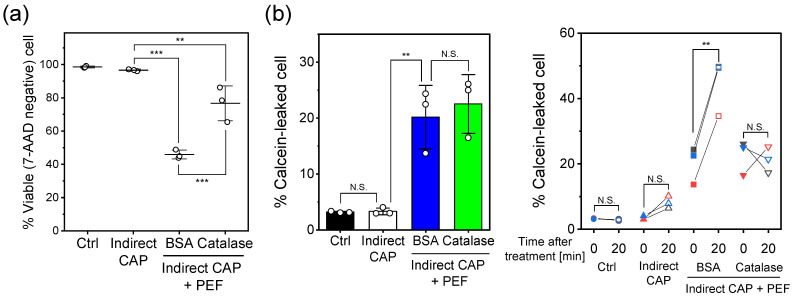
Catalase rescue experiment demonstrating the role of H_2_O_2_ in cytotoxicity and membrane damage. (**a**) Cell viability 24 h after treatment. (**b**) Membrane integrity assessed by calcein leakage. Left: the percentage of calcein-leaked cells immediately after treatment. Right: time-course analysis comparing calcein leakage at 0 and 20 min. Circles, Ctrl; triangles, Indirect CAP; squares, indirect +PEF (BSA); inverted triangles, indirect +PEF (Catalase). Each color represents an individual replicate from three independent experiments. Data are mean ± SD (n=3). Statistical significance was determined using one-way ANOVA followed by Tukey’s multiple comparison test (for panel a and immediate leakage) or paired *t*-test (for time-course analysis). ** p<0.01; *** p<0.001; N.S., not significant. Control (Ctrl): cells exposed to Ar gas flow for 3 min without plasma discharge.

To investigate the role of long-lived RONS, particularly H_2_O_2_, in the synergistic cytotoxicity and membrane damage, catalase rescue experiments were performed using the indirect CAP + PEF treatment model. Chemical analysis revealed that 3 min of Ar-APPJ irradiation generated 1100 ± 35 µM H_2_O_2_ in D-PBS (−) (n=3). Treatment with catalase (10 µg/mL) for 1 min reduced the H_2_O_2_ concentration to 7.7 ± 0.1 µM (n=3), confirming effective H_2_O_2_ scavenging. Figure 6 shows the results of catalase rescue experiments on cell viability and membrane integrity.

Figure 6a shows cell viability 24 h after treatment. Indirect CAP treatment alone maintained high viability (>95%), similar to control. Addition of BSA (control protein) to indirect CAP + PEF treatment resulted in approximately 46% viability, whereas catalase addition significantly improved viability to approximately 77% (p<0.001 vs. BSA), demonstrating that H_2_O_2_ plays a critical role in cytotoxicity.

Figure 6b shows membrane integrity assessed by calcein leakage. Immediately after treatment (Figure 6b, left), indirect CAP + PEF increased the percentage of calcein-leaked cells (approximately 20%), with no significant difference between BSA and catalase groups.

However, time-course analysis (Figure 6b, right) revealed striking differences in membrane resealing capacity. In the BSA group, the percentage of calcein-leaked cells increased significantly from 0 to 20 min (from ∼20% to ∼44%; p<0.01, paired *t*-test), indicating progressive membrane damage. In contrast, the catalase group showed minimal change over the same period (N.S.), demonstrating that H_2_O_2_ removal prevents progressive membrane damage. Control and indirect CAP alone groups maintained stable membrane integrity throughout the measurement period (N.S.).

These results indicate that H_2_O_2_ affects membrane integrity in a time-dependent manner following PEF-induced damage without directly creating immediate membrane pores.

## 3. Discussion

We investigated the enhanced cytotoxicity of combined CAP and PEF treatment in HeLa cells. While our previous report demonstrated cytotoxicity enhancement following combined treatment, the present study conducted a comparative analysis of treatment approaches to further elucidate the enhanced cytotoxicity. This study compared the cytotoxic effects of direct CAP irradiation with indirect treatment with CAP-treated liquid, both followed by PEF exposure.

Cell viability 24 h after treatment was assessed by 7-AAD staining. Figure 2 demonstrates that each single treatment maintained cell viability above 80%, providing suitable conditions for investigating synergistic cytotoxicity. However, both combination treatments (direct CAP + PEF and indirect CAP + PEF) resulted in significantly lower cell viability compared to the corresponding single treatments, indicating synergistic cytotoxic effects. Notably, both treatment groups that demonstrated significant cytotoxicity shared a common feature: HeLa cells were exposed to PEF in the presence of RONS-containing medium. Chemical analysis revealed that 3 min of Ar-APPJ irradiation generated 1100 ± 35 µM H_2_O_2_ in D-PBS (−). Furthermore, Figure 6a demonstrates that catalase addition, reducing H_2_O_2_ concentration to 7.7 ± 0.1 µM, prior to PEF significantly recovered cell viability. These findings suggest that PEF-induced membrane permeabilization facilitates intracellular delivery of long-lived RONS, particularly H_2_O_2_.

Comparison of the two combined treatments revealed that direct CAP + PEF showed significantly greater cytotoxicity than indirect CAP + PEF (Figure 2). This difference can be attributed to two factors: intracellular RONS levels and immediate membrane damage. Direct CAP alone induced significantly higher intracellular RONS levels than indirect CAP alone (approximately 2.06-fold vs. 1.64-fold; p<0.0001, Figure 3). We previously confirmed that intracellular RONS elevation by direct CAP irradiation is suppressed by NAC pretreatment under similar experimental conditions [16]. Furthermore, while both CAP treatments induced comparable levels of lipid peroxidation (Figure 4), direct CAP + PEF showed significantly greater immediate membrane damage than indirect CAP + PEF (24.4% vs. 15.4% calcein-leaked cells; p<0.01, Figure 5).

The comparable lipid peroxidation levels despite differential membrane damage represent an unexpected but reproducible observation confirmed through multiple independent experiments. PEF alone does not induce significant lipid peroxidation under comparable experimental conditions [40]. This finding suggests that short-lived RONS contribute to enhanced membrane permeabilization through mechanisms beyond lipid peroxidation alone. Several factors may explain this observation. First, the indirect CAP treatment conditions in this study (1.1 mM H_2_O_2_, 3-min incubation) represent a relatively strong oxidative stress that may have saturated lipid peroxidation responses. Under these conditions, the additional contribution of short-lived RONS present only in direct CAP treatment to lipid peroxidation may have been masked by the already substantial oxidative modifications induced by long-lived H_2_O_2_. This interpretation is consistent with the observation that both treatments showed approximately 1.4-fold increases in Liperfluo fluorescence (Figure 4b), suggesting that lipid peroxidation had reached a plateau level under our experimental conditions. Second, the enhanced immediate membrane damage observed with direct CAP + PEF (24.4% calcein-leaked cells) compared to indirect CAP + PEF (15.4% calcein-leaked cells) despite similar lipid peroxidation levels indicates that short-lived RONS must enhance membrane permeabilization through additional mechanisms. Our previous study demonstrated that direct CAP irradiation generates localized H_2_O_2_ accumulation at the liquid surface [41]. Additionally, Takajo et al. demonstrated that OH radicals are the primary species involved in CAP-induced liposome lipid peroxidation [42]. Short-lived RONS such as OH radicals and superoxide, which are present only during direct CAP irradiation, may induce oxidative damage to membrane proteins, potentially affecting membrane integrity and fluidity. These modifications could include: (1) oxidation of membrane proteins, which play critical roles in maintaining membrane integrity and could affect both electroporation sensitivity and resealing capacity; (2) alterations in membrane fluidity beyond those caused by lipid peroxidation, potentially through local disruption of lipid packing or changes in membrane tension; (3) potential modifications to the cytoskeleton-membrane interface, as interactions between the cytoskeleton and membrane are known to influence membrane mechanical properties and repair processes. The distinction between lipid peroxidation and functional membrane damage highlights an important consideration: while lipid peroxidation is a convenient marker of oxidative stress, it may not fully capture all oxidative modifications relevant to membrane electroporation sensitivity. Future studies examining protein oxidation, membrane fluidity changes, and cytoskeletal modifications could provide a more complete mechanistic understanding of how short-lived RONS enhance PEF-induced membrane permeabilization.

Time-course analysis of calcein leakage provides further mechanistic insights. While our previous report demonstrated that direct CAP + PEF enhanced calcein leakage compared to PEF alone, the temporal dynamics of membrane integrity disruption were not investigated. Both combination treatments showed progressive membrane damage from 0 to 20 min (Figure 5), with direct CAP + PEF increasing from 24.4% to 50.1% (p<0.01) and indirect CAP + PEF from 15.4% to 37.5% (p<0.05). The catalase rescue experiments (Figure 6b) provide definitive evidence for the mechanism of H_2_O_2_ action. Catalase treatment had no effect on immediate membrane damage (0 min), with both BSA and catalase groups showing comparable calcein leakage (∼20%, N.S., Figure 6b, left). This demonstrates that H_2_O_2_ does not directly create membrane pores. However, catalase dramatically prevented progressive membrane damage over time (BSA: 20.2% to 44.6%; p<0.01; catalase: no significant change, p=0.83, Figure 6b, right) and significantly improved cell viability from 46% to 77% (Figure 6a). These results indicate that H_2_O_2_ specifically inhibits membrane repair processes following PEF-induced electroporation, leading to sustained membrane disruption and cell death. This represents a fundamentally different mechanism from conventional H_2_O_2_ cytotoxicity, which relies on direct oxidative damage accumulation over extended periods.

H_2_O_2_ is extensively studied as a cytotoxic agent in oxidative stress research, with normal intracellular concentrations ranging from 1 to 700 nM [43]. While millimolar H_2_O_2_ concentrations can induce cell death, this typically requires prolonged exposure (hours to days), whereas short-term exposure (minutes) to even 1 mM H_2_O_2_ produces minimal cytotoxicity due to membrane barrier function and cellular antioxidant defenses. Although H_2_O_2_ combined with PEF has been reported for bacterial inactivation in food processing [44], where PEF facilitates H_2_O_2_ uptake, the present study reveals a fundamentally different mechanism in cancer cells: the presence of H_2_O_2_ at PEF application does not enhance uptake but specifically prevents membrane repair. This two-phase mechanism—immediate damage by PEF followed by H_2_O_2_-mediated repair inhibition—has not been previously reported to our knowledge and suggests novel therapeutic potential. The role of H_2_O_2_ in our CAP + PEF combination system shares mechanistic similarities with recent findings in food science, where H_2_O_2_ combined with PEF enhanced OH radical-mediated protein modification through sustained Fenton reactions [45]. However, our data suggest that in the context of cancer cell treatment, H_2_O_2_ primarily functions to inhibit membrane repair rather than directly facilitating electroporation. Laberie et al. reported that CAP promotes fibroblast migration persistence through the combined action of H_2_O_2_ and electric fields [46]. While their study focused on sublethal doses promoting cell migration, our findings reveal that at higher H_2_O_2_ concentrations combined with PEF-induced electroporation, the synergistic effect shifts from promoting migration to inducing cell death through membrane repair inhibition.

Chung et al. reported that plasma-treated PBS enhanced microsecond PEF-induced membrane electropermeabilization, attributing this effect to lipid oxidation that lowers the energy barrier for pore formation [39]. Vernier et al. similarly demonstrated that oxidative pretreatment of cells enhanced their susceptibility to electric field-induced permeabilization [47]. Molecular dynamics simulations by Yusupov et al. and Cui et al. also support this mechanism [48,49]. While our observation that CAP treatment enhances PEF-induced membrane permeabilization is consistent with these reports, our time-course analysis and catalase rescue experiments reveal an additional mechanism. Catalase treatment did not affect immediate membrane damage (Figure 6b, left), indicating that the presence of H_2_O_2_ at the moment of PEF application does not directly facilitate pore formation. Instead, H_2_O_2_ specifically inhibits membrane repair following PEF-induced damage, as evidenced by the complete prevention of progressive membrane disruption with catalase treatment (Figure 6b, right). Thus, our study identifies membrane repair inhibition as a distinct mechanism contributing to the synergistic cytotoxicity of combined CAP and PEF treatment.

**Figure 7:**
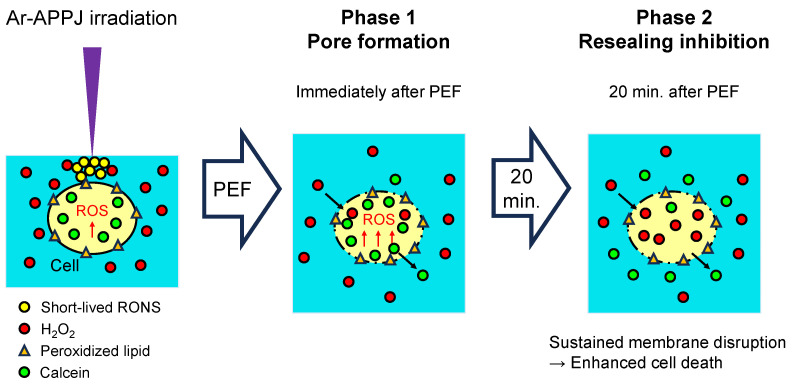
Schematic illustration of the proposed two-phase mechanism for synergistic cytotoxicity of combined CAP and PEF treatment. Following Ar-APPJ irradiation, cells are surrounded by CAP-generated reactive species including H_2_O_2_. In Phase 1, PEF induces membrane pore formation (electroporation), allowing calcein leakage and H_2_O_2_ influx. In Phase 2, H_2_O_2_ inhibits membrane resealing, resulting in sustained membrane disruption and progressive calcein leakage, ultimately leading to enhanced cell death. Black arrows indicate the direction of molecule movement across the membrane.

We investigated part of the synergistic cell death pathway using HeLa cells as a model system. Figure 7 illustrates the proposed two-phase mechanism for synergistic cytotoxicity of combined CAP and PEF treatment. Following Ar-APPJ irradiation, cells with peroxidized lipids and elevated intracellular RONS levels are surrounded by CAP-generated reactive species including H_2_O_2_. PEF then induces membrane pore formation, allowing calcein leakage and H_2_O_2_ influx. Subsequently, H_2_O_2_ inhibits membrane resealing, resulting in sustained membrane disruption and progressive calcein leakage, ultimately leading to enhanced cell death. However, several limitations should be noted. Given the heterogeneity in membrane composition and repair mechanisms across cell types, validation in additional cancer and normal cell lines is essential to establish generalizability. While we showed that H_2_O_2_ acts as a key reactive species through catalase rescue experiments, its concentration dependency and the roles of other long-lived RONS were not assessed. The molecular mechanisms underlying H_2_O_2_-mediated membrane repair inhibition remain incompletely characterized. Our finding that H_2_O_2_ specifically inhibits membrane resealing following PEF-induced electroporation raises the question of which type of cell death is induced. Recent studies have identified at least 12 distinct types of cell death, including apoptosis, necrosis, ferroptosis, and immunogenic cell death [50,51]. Classification of the cell death type observed in this study is required. All experiments were conducted in vitro; in vivo studies are necessary to assess therapeutic potential and safety. Finally, the 1-min interval between CAP and PEF treatments was based on our previous work but may require optimization for different applications.

## 4. Materials and Methods

### 4.1. Cell Culture

HeLa cells (RIKEN BioResource Research Center, Tsukuba, Japan) were cultured in Dulbecco’s modified Eagle’s medium (DMEM) with high glucose and phenol red (FUJIFILM Wako Pure Chemical, Osaka, Japan), supplemented with 10% fetal bovine serum (FBS; Thermo Fisher Scientific, Waltham, MA, USA), 100 units/mL penicillin, and 100 µg/mL streptomycin (PS; FUJIFILM Wako Pure Chemical) at 37 °C in a humidified atmosphere containing 5% CO_2_. Cells were grown in T75 flasks until reaching 50–70% confluence, then detached using 0.25% trypsin-EDTA (FUJIFILM Wako Pure Chemical), collected by centrifugation, and resuspended in Dulbecco’s modified phosphate-buffered saline without magnesium chloride and calcium chloride (D-PBS (−), FUJIFILM Wako Pure Chemical). Cell density was adjusted using a Countess 3 automated cell counter (Thermo Fisher Scientific) according to experimental requirements.

### 4.2. Ar-APPJ Generator, Plasma Characterization, and Irradiation Setup

Figure 1b,c show a schematic illustration of the CAP irradiation setup and a photograph of the Ar-APPJ, respectively. The Ar-APPJ generator used in this study was identical to that described in our previous publications [17,40]. Briefly, the generator consisted of a quartz glass tube with an inner/outer diameter of 1.5/2.1 mm and two copper tape electrodes (10 mm width) spaced 5 mm apart. The upper electrode was powered while the lower electrode was grounded. The gap between the grounded electrode and the nozzle was 10 mm.

The applied voltage was an 18 kV_p-p_ sinusoidal waveform at 18 kHz, monitored using an oscilloscope (TDS2014; Tektronix, Beaverton, OR, USA) with a high-voltage probe (P6015A; Tektronix). The high-voltage generation system comprised a DC power supply (PK80H; Matsusada Precision, Kusatsu, Shiga, Japan) connected to a neon-sign inverter transformer (ALPHA NEON M-5; Lecip, Motosu, Gifu, Japan). Discharge power was determined to be less than 1 W using the Lissajous method. The non-thermal nature of the Ar-APPJ was confirmed by liquid temperature measurements: the temperature of D-PBS (−) after 3 min of Ar-APPJ irradiation at 10 mm treatment distance was measured using a digital thermometer and found to be comparable to that under Ar gas flow without plasma discharge, remaining at approximately room temperature.

Argon (>99.99 vol.% purity) served as the carrier gas at a flow rate of 0.7 L/min, regulated using a float-type flow meter (Kofloc, Nagoya, Aichi, Japan).

The reactive species composition of this Ar-APPJ source is relevant to the interpretation of results in this study. Optical emission spectroscopy of the plasma at 5 mm below the nozzle revealed characteristic emission lines of Ar (690–850 nm), the N_2_ second positive system (330–400 nm), and OH radicals (306–309 nm), confirming the generation of both oxygen- and nitrogen-based reactive species through plasma–air interaction at the liquid surface [40]. Under the experimental conditions employed in this study (3 min irradiation of 300 µL D-PBS (−) at 10 mm treatment distance), the Ar-APPJ generates 1100 ± 35 µM H_2_O_2_ as the dominant long-lived RONS species, as quantified in Section 2.5. Short-lived species including OH radicals and superoxide are present exclusively during direct irradiation and decay within microseconds, providing the mechanistic basis for the experimental distinction between direct and indirect CAP treatment in this study.

### 4.3. PEF Generator

PEF was generated using a commercially available electroporator (NEPA21, Nepa Gene, Chiba, Japan). Figure 1d shows the waveform. PEF consisted of two consecutive pulses with the following parameters: pulse duration of 5 ms, pulse interval of 50 ms, maximum electric field strength of 1.0 kV/cm, and decay ratio of 10%. For PEF treatment, 100 µL of cell suspension was transferred into 2 mm-gap electroporation cuvettes (Nepa Gene).

### 4.4. Ar-APPJ Irradiation and PEF Application to HeLa Cells

HeLa cells were treated using either direct or indirect CAP irradiation, both followed by PEF application (Figure 1a). For direct CAP treatment, a 300 µL aliquot of cell suspension (1.0 × 10^7^ cells/mL) was added to one well of a 96-well cell culture plate. The distance between the nozzle and the cell suspension surface was maintained at 10 mm. Cell suspensions were irradiated with Ar-APPJ for 3 min at room temperature. Following treatment, 100 µL of cell suspension was transferred to an electroporation cuvette. The time interval between Ar-APPJ and PEF treatments was maintained at 1 min. Remaining cell suspension was used as direct CAP alone.

For indirect CAP treatment, a 300 µL aliquot of D-PBS (−) was added to one well of a 96-well cell culture plate, then irradiated with Ar-APPJ for 3 min, as described above. A total of 0.3 × 10^7^ HeLa cells were suspended in 300 µL of the Ar-APPJ-irradiated D-PBS (−) and incubated for 3 min at room temperature. Following incubation, the cell suspension was treated with PEF. Remaining cell suspension was used as indirect CAP alone.

Control samples were exposed to argon gas flow for 3 min without plasma discharge. A total of 100 µL of cell suspension was subjected to PEF (PEF alone). Remaining cell suspension was used as untreated control.

### 4.5. Chemical Analysis of H_2_O_2_

H_2_O_2_ concentration in plasma-treated D-PBS (−) was quantified using the Pierce Quantitative Peroxide Assay Kit (Thermo Fisher Scientific) with a multimode microplate reader (Varioskan Flash, Thermo Fisher Scientific) according to the manufacturer’s instructions. To verify catalase activity, H_2_O_2_ concentration was also measured after 1-min incubation with catalase (10 µg/mL) at room temperature.

### 4.6. Cell Viability

After treatment, cells were resuspended in 6 mL of DMEM supplemented with 10% FBS and PS, transferred to a 6-well cell culture plate, and cultured for 24 h at 37 °C in a humidified 5% CO_2_ incubator. Both floating cells in the medium and adherent cells detached by trypsinization were harvested by centrifugation. After resuspension in 500 µL of D-PBS (−), 7-amino-actinomycin D (7-AAD; Beckman Coulter, Brea, CA, USA) was added to stain dead cells. After incubation with 7-AAD for 20 min at room temperature, cell viability was analyzed by flow cytometry (CytoFLEX, Beckman Coulter).

### 4.7. Intracellular RONS Measurement

Intracellular RONS levels were quantified using photo-oxidation-resistant DCFH-DA dye (Dojindo, Kumamoto, Japan) according to the manufacturer’s instructions. Briefly, cells were incubated with 10 µM DCFH-DA working solution for 30 min at 37 °C. After incubation, the probe-loaded cells were washed with D-PBS (−) twice, trypsinized, resuspended in D-PBS (−), and adjusted to 1.0 × 10^7^ cells/mL. The cell suspension was subjected to CAP and/or PEF treatment, and fluorescence intensity of the cells was immediately measured using flow cytometry.

### 4.8. Lipid Peroxidation Measurement

Lipid peroxidation was assessed using the Liperfluo reagent (Dojindo) according to the manufacturer’s instructions and our previous report [40]. HeLa cells were incubated with 5 µM Liperfluo for 30 min at 37 °C in a humidified 5% CO_2_ atmosphere. Following incubation, cells were washed with D-PBS (−), detached by trypsinization, and resuspended in D-PBS (−). The cell suspension was then subjected to the treatment protocol outlined in Section 4.4. Fluorescence intensity was subsequently measured by flow cytometry.

### 4.9. Calcein Leakage Measurement

Membrane integrity was assessed using calcein-AM, a cell-permeant fluorescent dye that is converted to cell-impermeable calcein upon intracellular esterase cleavage. This assay was performed as previously described with some modifications [40,52]. HeLa cells were loaded with 0.5 µM calcein-AM (Dojindo) in D-PBS (−) for 30 min at 37 °C. After loading, cells were harvested by centrifugation and resuspended in D-PBS (−). Following cell concentration adjustment, the suspension was subjected to the treatment protocol as described in Section 4.4. A 100 µL aliquot of the treated cell suspension was then mixed with 500 µL of D-PBS (−), and fluorescence intensity was measured by flow cytometry immediately after treatment. Subsequently, the samples were incubated for 20 min at 37 °C and measured again to evaluate calcein retention and membrane resealing capacity.

### 4.10. Catalase Rescue Experiments

To assess the role of H_2_O_2_, a representative long-lived RONS generated by CAP treatment, in cytotoxicity and membrane damage, catalase rescue experiments were performed using the indirect CAP + PEF treatment model. Following indirect CAP treatment (as described in Section 4.4), either catalase (10 µg/mL; FUJIFILM Wako Pure Chemical) or bovine serum albumin (BSA, 10 µg/mL; Thermo Fisher Scientific) as a control protein was added to the indirect CAP-treated cell suspension. Catalase concentration was determined based on our previous paper [41]. After a 1-min incubation at room temperature, PEF was applied. Cell viability was assessed by 7-AAD staining after 24 h of culture, and calcein leakage was measured immediately and 20 min after treatment as described in Section 4.6 and Section 4.9, respectively.

### 4.11. Flow Cytometry

Fluorescence intensity of individual cells was measured using a CytoFLEX flow cytometer (Beckman Coulter) equipped with CytExpert Version 2.6 data acquisition software (Beckman Coulter). Prior to flow cytometric analysis, samples were filtered through a 35-µm nylon cell strainer (Corning, NY, USA) to remove cellular debris. Data were acquired from a minimum of 10,000 events for each experimental condition. Data analysis was performed using Kaluza Analysis 2.1 software (Beckman Coulter).

### 4.12. Statistical Analysis

All experiments were performed in three independent replicates. Data are presented as mean ± standard deviation (SD). Statistical significance was assessed using one-way ANOVA followed by Tukey’s multiple comparison test or paired *t*-test. Paired *t*-tests were used for comparisons within the same sample group (e.g., time-course analyses), whereas one-way ANOVA followed by Tukey’s test was used for comparisons among independent groups. All statistical analyses were performed using OriginPro 2026 (OriginLab Corporation, Northampton, MA, USA). The raw data are available in Tables S1–S7.

## Data Availability

The original contributions presented in this study are included in the article. Further inquiries can be directed to the corresponding author.

## Supplementary Materials

The following supporting information can be downloaded at: https://www.mdpi.com/article/10.3390/ijms27062700/s1.
