# Supplementary material for: Hydrogen Peroxide-Mediated Inhibition of Membrane Resealing Drives Synergistic Cytotoxicity of Combined Cold Atmospheric Plasma and Pulsed Electric Field Treatment
Source: Int J Mol Sci. 2026 Mar 16;27(6):2700. doi: 10.3390/ijms27062700 (PMC13026970; doi:10.3390/ijms27062700)
Supplement: Supplementary file 1 [file ijms-27-02700-s001.zip › ijms-4146195-supplementary.pdf]

Supporting materials

Supplementary Table S1. Cell viability (7-AAD staining, Figure 2(b))

Treatment	Ctrl	Direct CAP	Indirect CAP	PEF	Direct + PEF	Indirect + PEF
Rep. #1 (%)	96.9	81.2	88.1	87.4	17.0	38.3
Rep. #2 (%)	99.1	87.1	88.7	84.4	18.4	34.7
Rep. #3 (%)	97.1	81.6	88.9	89.1	15.9	26.1
Mean (%)	97.7	83.3	88.6	87.0	17.1	33.0
SD (%)	1.2	3.3	0.4	2.4	1.3	6.3

Supplementary Table S2. Intracellular RONS
(relative DCFH fluorescence, Figure 3(b))

Treatment	Ctrl	PEF	Direct CAP	Direct + PEF	Indirect CAP	Indirect + PEF
Rep. #1 (Fold)	0.99	0.97	2.08	2.44	1.6	1.56
Rep. #2 (Fold)	0.99	0.97	2.06	2.38	1.75	1.55
Rep. #3 (Fold)	1.02	0.98	2.03	2.30	1.59	1.50
Mean (Fold)	1.00	0.97	2.06	2.37	1.64	1.54
SD (Fold)	0.018	0.004	0.022	0.069	0.089	0.031

Supplementary Table S3. Lipid peroxidation
(relative Liperfluo fluorescence, Figure 4(b))

Treatment	Ctrl	Direct CAP	Indirect CAP
Rep. #1 (Fold)	1.00	1.40	1.43
Rep. #2 (Fold)	1.00	1.37	1.41
Rep. #3 (Fold)	1.00	1.36	1.34
Mean (Fold)	1.00	1.38	1.39
SD (Fold)	0.001	0.021	0.045

Supplementary Table S4. The percentage of calcein-leaked cells (0 min, Figure 5(b))

Treatment	Ctrl	Direct + PEF	Indirect + PEF
Rep. #1 (%)	2.8	21.2	15.8
Rep. #2 (%)	3.6	24.9	14.7
Rep. #3 (%)	3.4	27.1	15.7
Mean (%)	3.3	24.4	15.4
SD (%)	0.4	3.0	0.6

Supplementary Table S5. The percentage of calcein-leaked cells (time course, Figure 5(c))

Treatment	Ctrl		Direct CAP + PEF		Indirect CAP + PEF	
	0 min	20 min	0 min	20 min	0 min	20 min
Rep. #1 (%)	2.8	2.7	21.2	49.2	15.8	46.0
Rep. #2 (%)	3.6	2.5	24.9	51.5	14.7	33.5
Rep. #3 (%)	3.4	2.7	27.1	49.7	15.7	32.9
Mean (%)	3.3	2.6	24.4	50.1	15.4	37.5
SD (%)	0.4	0.1	3.0	1.2	0.6	7.4

Supplementary Table S6. Catalase rescue (cell viability, Figure 6(a))

Treatment	Ctrl	Indirect CAP	Indirect CAP + PEF	
			BSA	Catalase
Rep. #1 (%)	98.0	96.0	43.9	65.4
Rep. #2 (%)	99.1	97.1	48.8	78.4
Rep. #3 (%)	98.4	96.7	44.9	86.2
Mean (%)	98.5	96.6	45.9	76.7
SD (%)	0.6	0.6	2.6	10.5

Supplementary Table S7. Catalase rescue
(the percentage of calcein-leaked cells, Figure 6(b))

Treatment	Ctrl		Indirect CAP		Indirect CAP + PEF			
					BSA		Catalase	
	0 min	20 min	0 min	20 min	0 min	20 min	0 min	20 min
Rep. #1 (%)	3.1	3.0	3.0	6.4	24.4	49.8	26.1	17.2
Rep. #2 (%)	3.3	2.6	3.0	10.1	13.7	34.6	16.5	25.2
Rep. #3 (%)	3.1	2.7	4.0	7.8	22.5	49.4	25.0	21.4
Mean (%)	3.2	2.8	3.3	8.1	20.2	44.6	22.5	21.3
SD (%)	0.1	0.2	0.6	1.9	5.7	8.6	5.3	4.0
